# Neuropeptide Localization in *Lymnaea stagnalis*: From the Central Nervous System to Subcellular Compartments

**DOI:** 10.3389/fnmol.2021.670303

**Published:** 2021-05-20

**Authors:** Ellen A. Wood, Sylwia A. Stopka, Linwen Zhang, Sara Mattson, Gabor Maasz, Zsolt Pirger, Akos Vertes

**Affiliations:** ^1^Department of Chemistry, The George Washington University, Washington, DC, United States; ^2^Department of Neurosurgery, Brigham and Women’s Hospital, Harvard Medical School, Boston, MA, United States; ^3^Balaton Limnological Research Institute, Eötvös Loránd Research Network (ELKH), Tihany, Hungary; ^4^Soós Ernő Research and Development Center, University of Pannonia, Nagykanizsa, Hungary

**Keywords:** *Lymnaea stagnalis*, mass spectrometry, neuropeptide, central nervous system, single neuron analysis

## Abstract

Due to the relatively small number of neurons (few tens of thousands), the well-established multipurpose model organism *Lymnaea stagnalis*, great pond snail, has been extensively used to study the functioning of the nervous system. Unlike the more complex brains of higher organisms, *L. stagnalis* has a relatively simple central nervous system (CNS) with well-defined circuits (e.g., feeding, locomotion, learning, and memory) and identified individual neurons (e.g., cerebral giant cell, CGC), which generate behavioral patterns. Accumulating information from electrophysiological experiments maps the network of neuronal connections and the neuronal circuits responsible for basic life functions. Chemical signaling between synaptic-coupled neurons is underpinned by neurotransmitters and neuropeptides. This review looks at the rapidly expanding contributions of mass spectrometry (MS) to neuropeptide discovery and identification at different granularity of CNS organization. Abundances and distributions of neuropeptides in the whole CNS, eleven interconnected ganglia, neuronal clusters, single neurons, and subcellular compartments are captured by MS imaging and single cell analysis techniques. Combining neuropeptide expression and electrophysiological data, and aided by genomic and transcriptomic information, the molecular basis of CNS-controlled biological functions is increasingly revealed.

## Introduction

### Neuropeptides in *Lymnaea stagnalis*

**FIGURE 1:**
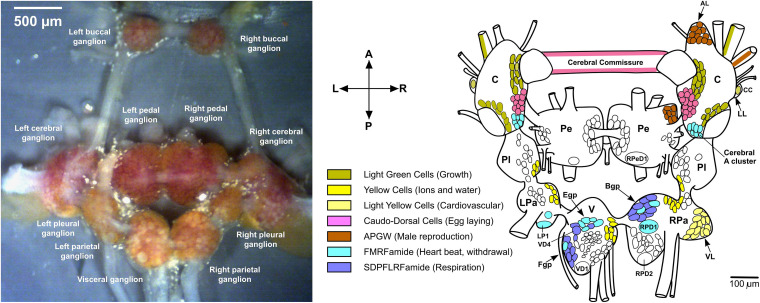
Dissected *Lymnaea* CNS and distribution of some neuropeptides. **(left)** Dissected *L. stagnalis* CNS (Zhang et al., 2018). **(right)** Localization of some peptides to identified neurons (Benjamin and Kemenes, 2013). The anterior lobe (AL) hosts some APGW neurons. Abbreviations: C, cerebral ganglion; CC, Canopy Cell; B/Egp, B/E group; LL, lateral lobe; LP1, left parietal 1; LPa, left parietal ganglion; Pe, pedal ganglion; Pl, pleural ganglion; RPa, right parietal ganglion; RPD1/2, right parietal dorsal 1/2; RPeD1, right pedal dorsal 1; V, visceral ganglion; VD1, visceral dorsal 1; VD4, visceral dorsal 4; and VL ventral lobe.

*Lymnaea stagnalis*, also known as the great pond snail, is an excellent model organism used for research in neuroscience, aging, ecotoxicology, parasite-host interactions, and evolution and development (Jimenez et al., 2004; Hoek et al., 2005; Benjamin, 2008; Pirger et al., 2014; Fodor et al., 2020a,b, 2021). Specifically, the central nervous system (CNS) of *L. stagnalis* is highly characterized at multiple levels. It is composed of eleven interconnected ganglia, each containing their own unique sub-populations of large and brightly colored neurons with specific functions and expressing a set of neuropeptides, as shown in Figure 1 (Benjamin and Kemenes, 2013; Rivi et al., 2020). In comparison to vertebrates, the CNS of *L. stagnalis* is considered more accessible because of the vastly lower number of neurons (∼20,000 compared to, e.g., 86,060,000,000 for humans; Azevedo et al., 2009), and partially elucidated neuronal circuits for specific functions. Functioning of the CNS, ganglia, neuronal circuits, and individual neurons relies in part on spatial and temporal variations in their chemical composition. From the early 1980s, neuropeptides have become a focal point for research due to their direct role in modulating neuronal circuit functions (that also occurs in the vertebrate nervous system) within the CNS (Schot et al., 1981; Patel et al., 2005).

The sensory system of *L. stagnalis* responds to a variety of external stimuli (e.g., a chemical stimulus – smell), signaling a modulatory response to the central pattern generator (CPG) networks that initiate the motor system (e.g., feeding moto-neurons in buccal ganglia), and induces an observable physical response (e.g., feeding behavior – radula movement). Once a stimulus is received in the sensory system, a signal is sent to the modulatory system where neuropeptides convey the signal, further cascading it to the motor system (Kemenes et al., 2001). Extensive research has revealed biochemical processes associated with external stimuli and intracellular components such as learning and memory systems, whole-body withdrawal, heartbeat, feeding, and respiratory motor circuits (Buckett et al., 1990a,b; Brierley et al., 1997; Fulton et al., 2005). For example, strong correlation between the abundance of insulin in the CNS and long-term memory has been established (Kojima et al., 2015; Totani et al., 2019). Understanding the correlation between an introduced external stimulus, such as a tactile, photoreceptive, or food-related excitation, and an observed behavior of *L. stagnalis* in response, has been the driving force for continued research into the molecular profiling of CNS circuits. By identifying the neuropeptide profiles and localization in the CNS, a better consensus on the correlation between their composition, biochemical processes, and physiology can be achieved.

With the recent availability of the unannotated draft genome for *L. stagnalis* (Stevens et al., 2016), and the emerging transcript information (see the four major transcriptome databases), finding the sequences for neuropeptide precursor proteins (prohormones) is accelerating (Davison and Blaxter, 2005; Feng et al., 2009; Bouetard et al., 2012; Sadamoto et al., 2012). Comprehensive inventories of prohormones and predicted neuropeptides for other mollusks are being constructed. For example, for the gray garden slug (*Deroceras reticulatum*), 65 neuropeptide precursor genes and more than 330 neuropeptides were putatively identified (Ahn et al., 2017), or for the common the Roman snail (*Helix pomatia*), 12 neuropeptide precursor genes and more than 100 neuropeptides (clustered in 26 distinct peptide families) were predicted (Kiss and Pirger, 2006). Similar to these, approximately 100 (neuro)peptides have been identified in *L. stagnalis* so far (Benjamin and Kemenes, 2020) encoded by genes involved in various regulatory processes. The collection of 98 *L. stagnalis* neuropeptides listed in Supplementary Table 1, is assembled from other data collections (Wang et al., 2015) and the literature. Most of them are clustered according to their prohormone (protein precursor). In addition, families based on sequence homologies and similarity of function are also indicated. The latter include the FMRFamide, FMRFamide-related, small cardioactive peptides (SCP), insulin, molluscan ELH, myomodulin, LYC, α, FxRI, LYCEP, LIP, and vasopressin/oxytocin families.

Initially, the distribution of highly expressed neuropeptides was explored by oligonucleotide probes that hybridized to the mRNA coding for the relevant segments of the prohormone and carried a fluorescent tag. For example, such studies indicated that the APGW-amide tetrapeptide was highly expressed in the right anterior lobe of the cerebral ganglia (Smit et al., 1992). An alternative approach relied on immunocytochemistry based on antisera raised against the peptides of interest (Santama et al., 1994a). Systematic studies revealed expression level variations with cellular resolution.

**FIGURE 2:**
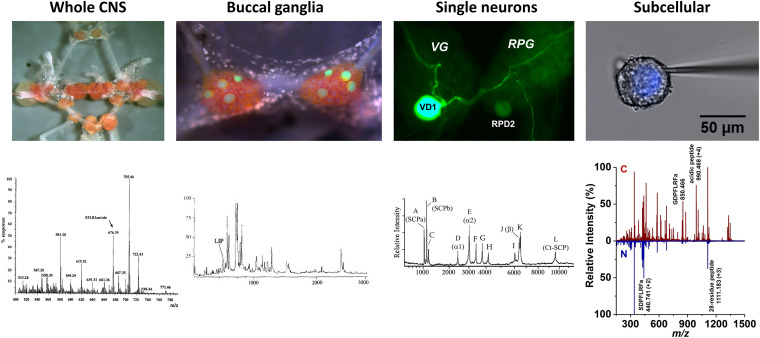
Neuropeptide localization and mass spectra at four levels of organization. Images of whole CNS, buccal ganglia with identified feeding motoneurons, B1–B4, highlighted by lucifer-yellow intracellular filling, single visceral dorsal 1 (VD1) and right parietal dorsal 2 (RPD2) neurons with intracellular filling, and nucleus of Type 2 Fgp neuron and corresponding mass spectra (Santama et al., 1995, Li et al., 1996, Jimenez et al., 2006, and Zhang et al., 2018), respectively, revealed the expression of specific peptides.

In the early 1990s, single cell level molecular analysis was enabled by the large size of some neurons (>50 μm) readily accessible in *L. stagnalis* and the emerging high spatial resolution and sensitivity of mass spectrometry (MS). Matrix-assisted laser desorption ionization (MALDI) MS made possible some of the earliest neuropeptide analysis experiments in single neurons (Vanveelen et al., 1993; Jimenez et al., 1994; Kellett et al., 1996). This extended molecular profiling, neuropeptide discovery and identification from whole CNS samples to single neurons and ultimately cellular compartments (Rubakhin et al., 2000, 2003; Altelaar et al., 2005; McDonnell et al., 2005). Multilevel understanding of neuropeptide distributions from the whole CNS to subcellular detail is critical to provide new insight into biochemical processes associated with the functioning CNS (Buckett et al., 1990b; Fodor et al., 2020a). Following the multilevel approach (from behavior to circuits, and molecules) in *L. stagnalis* research, Figure 2 shows the four levels of organization in the CNS, the corresponding mass spectra, and the localization of some neuropeptides.

### Advances in Mass Spectrometry Bring Neuropeptide Localization in Focus

The analysis of neuropeptides within the CNS of *L. stagnalis* has been an area of interest for decades, and new methodologies to improve detection are continually being introduced. Early neuropeptide research used a variety of techniques, such as the hybridization to DNA/RNA with exons coding for the targeted peptides, cloning, fluorescence, and immunohistochemistry (IHC) (Linacre et al., 1990; Bright et al., 1993; Santama et al., 1993). For example, the early recognition of molluscan insulin-related peptides was based on screening of a large cDNA library that revealed coding for preproinsulin (Smit et al., 1991). Neuropeptide discovery by some of these techniques was a time-consuming endeavor and required large sample sizes. For example, to discern neuropeptide primary structures in *L. stagnalis* required up to 500 ganglia and 1 million large neurons (Li et al., 1994a).

Initially, MS was an auxiliary method to indicate the expression of peptides predicted from cDNA sequences and partially verified by IHC (Santama et al., 1994b). The introduction of MALDI-MS to invertebrate neuroscience dramatically decreased the amount of sample required for peptide analysis and rapidly reached single neuron resolution (Jimenez et al., 1994). Due to instrumental limitations, however, these MALDI mass spectra only reported the nominal mass of the predicted peptide, in some cases only with a few Dalton (Da) accuracy.

With the application of tandem MS and improvements in instrument characteristics in the following decade, MS became a workhorse for the detection, identification, and quantitation of neuropeptides in a variety of biological samples, including invertebrates (Chaurand et al., 1999; Hummon et al., 2003, 2006). Improved mass accuracy and tandem MS enhanced the identification of neuropeptides, whereas MS imaging (MSI) helped to determine their distributions and localization in the organism (Caprioli et al., 1997). The ability to elucidate the primary structure of novel neuropeptides, and to reveal their localization in the CNS made MS an important tool for molecular exploration in *L. stagnalis*. In some cases, peptide sequencing by tandem MS pinpointed potential errors in the cDNA sequence and demonstrated the expression of an unexpected peptide in a particular cell type (Zhang et al., 2018).

Early on, tandem MS techniques helped with the de novo sequencing of the detected peptides to establish their relationship with the underlying genes, transcripts, and precursor proteins (Dreisewerd et al., 1997; Li et al., 1997; Jimenez et al., 1998). To reduce the complexity of tissue extracts, high-performance liquid chromatography (HPLC) was combined with MS techniques (Dewith and Vanderschors, 1992; Santama et al., 1995). However, for volume limited samples, i.e., single cells and subcellular compartments, HPLC is not suitable. Instead, the application of ion mobility separation (IMS) is being introduced (Zhang et al., 2018). Combining IMS with MS reduces the sample volume requirement for separation, and dramatically shortens the separation times from 10s of minutes to the ms timescale.

Neuropeptide imaging by MSI techniques became of interest to further elucidate neuropeptide distributions and colocalizations within the CNS. They include MALDI-MSI and matrix-enhanced secondary ion mass spectrometry (ME-SIMS) that are highly sensitive and used to spatially map neuropeptides within the CNS of *L. stagnalis* (Altelaar et al., 2005; McDonnell et al., 2005; Chen and Li, 2010). MSI techniques, including MADLI-MSI and ME-SIMS, have expanded the analysis of neuropeptides by tracking ions of interest and showing their distribution throughout the sample, which assists in determining localization within the CNS. As the name suggests, ME-SIMS combines the use of matrix for sample preparation and the imaging capabilities of SIMS. An MS image is produced by sputtering the surface of the sample with a focused primary ion beam, then collecting and analyzing ejected secondary ions (Altelaar et al., 2005; McDonnell et al., 2005; Chen and Li, 2010). The sensitivity of SIMS for higher mass molecules is improved by the addition of a matrix, thus the motivation behind the combination of techniques for ME-SIMS is to combine the high mass capabilities of MALDI with the high spatial resolution of SIMS (Altelaar et al., 2005; McDonnell et al., 2005; Chen and Li, 2010). With the use of ME-SIMS, high resolution molecular ion maps of the neuropeptide APGW were created, thus finding localization predominantly in the right anterior lobe of the right cerebral ganglia. Neurons in this region are known to regulate male copulation behavior in *L. stagnalis* (Altelaar et al., 2005). These findings help to correlate the localization of neuropeptides in the CNS to their biological role. Upon new imaging findings and improved instrumentation, these techniques have shown that direct molecular imaging of *L. stagnalis* nervous tissue is possible and that the continued development of MS techniques will further accelerate neuropeptide profiling.

Here we present an account of recent developments in the analysis of neuropeptides by MS in the nervous system of *L. stagnalis* with granularity spanning from the whole CNS to subcellular details in identified cells.

## Whole CNS Studies

Early research using *L. stagnalis* focused on neuropeptide identifications and distributions within the entire CNS. Non-MS techniques, such as immunocytochemistry and intracellular dye filling reported on the spatial distributions of multiple neuropeptide families including FMRFamide related, myomodulin, and SCP, whereas DNA/RNA hybridization and DNA extractions helped with predicting the amino acid sequence. The ability to link biological functions to the presence of these peptides in *L. stagnalis*, e.g., heart modulation, feeding behavior, and male copulation, made them attractive for early CNS analyses (Ebberink et al., 1987; Santama et al., 1994a,b; Benjamin, 2008).

Leading up to MS analysis of *L. stagnalis*, other mollusk model organisms, for example, *Aplysia californica*, were studied for neuropeptide identification, using both non-MS and MS techniques (Weiss et al., 1984; Sweedler et al., 2002). The FMRFamide peptides and the feeding circuit activating peptides (FCAPs) had been previously identified in the nervous system of *A. californica*, thus raising the possibility of their presence or the presence of their homologues in *L. stagnalis* due to similarities between the two invertebrates. The FMRFamide related peptide family was linked to heart modulation (Benjamin, 2008; Fodor et al., 2020a), making it of interest for early neuropeptide research. This neuropeptide class consisted of tetrapeptides (FMRF/FLRF) and heptapeptides (SDPFLRF/GDPFLRF) (Ebberink et al., 1987). In addition, other neuropeptide classes of biological importance, such as myomodulin and SCP, previously found in other mollusks (Kiss and Pirger, 2006), were also studied in *L. stagnalis* to further explore their role in gut modulation and male copulation. Using IHC, the distributions of these peptides throughout the CNS, e.g., myomodulin A (PMSMLRL) and SCP (SGYLAFPRM), were tentatively determined (Santama et al., 1994a,b; Benjamin and Kemenes, 2013).

Mass spectrometry techniques validated previous discoveries, as well as identified novel neuropeptides. With the addition of MS techniques, neuropeptide identifications expanded into a variety of functionally identified classes (see Supplementary Table 1), as well as the elucidation of new unlabeled neuropeptides (Fodor et al., 2020a). For example, analysis was done on multiple neuropeptides encoded on the FMRFamide locus of the snail. By combining HPLC and continuous-flow fast atom bombardment MS, three novel peptides, EFLRlamide, pQFYRlamide, and pQFLRlamide, were identified within an extract of 20 CNS samples (Santama et al., 1995). Additionally, MS analysis of the CNS tentatively recognized three novel peptides SKPYMRFamide, HDYMRFamide, and SSFPRYamide (Kellett et al., 1994). Taken together with earlier work, these findings expanded the biologically important FMRFamide neuropeptide class.

**FIGURE 3:**
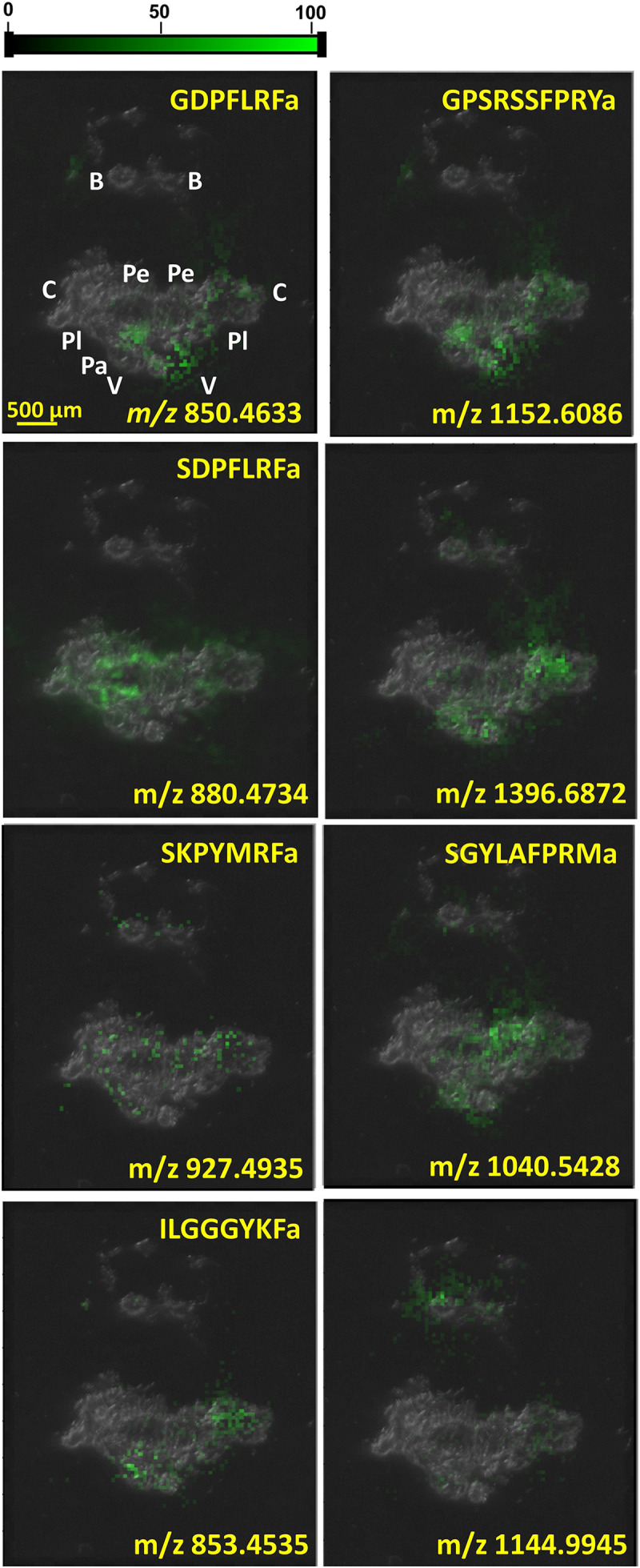
Neuropeptide distributions in CNS based on MALDI-MSI overlaid on top of optical microscope image of CNS section. Several other peptides were uniformly distributed throughout the whole CNS, whereas the six known and two unknown peptides depicted above were significantly more abundant in specific ganglia.

As neuropeptide classes, like FMRFamide-related peptides, were being explored, interest in their distribution within the CNS was growing. IHC indicated the preferential expression of particular neuropeptides in certain cell types (see, e.g., Santama et al., 1994b). In the coming years, this motivated the implementation of MSI techniques for molecular imaging and the targeted analysis of individual ganglia and neuronal clusters. Figure 3 shows the distributions of six identified and two unidentified neuropeptides, GDPFLRFamide (*m/z* 850.4633), ILGGGYKFamide (*m/z* 853.4535), SDPFLRFamide (*m/z* 880.4734), SKPYMRFamide (*m/z* 927.4935), SGYLAFPRMamide (*m/z* 1040.5428), *m/z* 1144.9945, GPSRSSFPRYamide (*m/z* 1152.6086), and *m/z* 1396.6872, in the CNS as they are reported by MALDI-MSI.

## Individual Ganglia and Neuronal Clusters

The CNS of *L. stagnalis* contains 11 interconnected ganglia, each with their own unique subpopulations of neurons on the surface of the ganglia. Previous whole CNS neuropeptide analyses have given rise to identification and validation of specific neuropeptide classes with important biological functions. Analysis of individual ganglia revealed neuropeptide localization within the CNS in greater detail.

Molecular components linked to reproduction in *L. stagnalis* have been of interest due to the hermaphroditic behavior and the abundance of correlated neuropeptide messengers (El Filali et al., 2006; Koene, 2010). Research has located multiple ganglia, numerous neuron clusters, and diverse neuropeptides associated with both male and female reproduction. Peptides involved in male copulation include APGW, conopressin, *C*-terminally located anterior lobe peptide, DEILSR, EFLRI, FLRF, FVRIs, G/SDPFLRF, gonadotropin releasing hormone, LIP A, B, and C, Lymnaea neuropeptide Y, myomodulins, pedal peptide, pQFYRI, and SEEPLY (Koene, 2010). In females, HF(FH)FYGPYDVFQRDV, 5895 Da peptide, amino terminal peptide, calfluxin, carboxyl terminal peptide, CDCH, α-CDCP, β1-CDCP, β2-CDCP, β3-CDCP, dorsal body hormone, LFRF, gonadotropin releasing hormone, γ-, δ-, and ε-peptides were found to be involved (Koene, 2010). For example, specific neuron clusters have been located in the right cerebral ganglia, specifically in the ventral and anterior lobe, which have a role in the male copulation behavior, and contain APGW (De Boer et al., 1997; Li et al., 1997). Another neuropeptide of interest due to its role in male copulation is myomodulin A (Li et al., 1994b). One of the first studies for myomodulin A using MS aimed to validate the presence of this neuropeptide, found in *A. californica*, also in *L. stagnalis*. After establishing its presence, IHC and MS suggested that myomodulin A is synthesized in neurons of the ventral lobe, located in the right cerebral ganglion (Li et al., 1994b). MS analysis of the right cerebral ganglion, specifically the cluster of neurons in the ventral lobe, revealed two more unique neuropeptides, SCP A and SCP B (Dreisewerd et al., 1997). It was also determined that none of these neuropeptides were found in the anterior lobe of this ganglion, further supporting a specific localization within a cluster of neurons involved in male copulation.

Previously unidentified neuropeptide classes and their localization were discovered using MALDI-MS. The neuropeptide, GAPRFVamide, once labeled as *L. stagnalis* inhibitory peptide (LIP), was found in the buccal ganglia (BG), and the ventral lobe, along with two other -FVamide neuropeptides (LIP B and C). GAPRFVamide is now labeled as LIP A and is included within the LIP neuropeptide class linked to the inhibitory effect on the spontaneous contraction and relaxation cycle in male copulation (Smit et al., 2003). Analysis of the neuropeptide APGW, and the myomodulin, SCP, and LIP neuropeptide classes within the specific ventral lobe cluster of neurons verified that localization of neuropeptides could correlate with their biological roles (van Tol-Steye et al., 1999).

MALDI-MSI of the whole CNS in Figure 3 indicates the localization of some neuropeptides to specific ganglia and neuronal clusters. The neuropeptide GPSRRSSFPRYamide is localized in the left parietal, visceral, and cerebral ganglia, which correlates with its diverse functions. Neuropeptide SCP A (SGYLAFPRMamide) is detected in the right pedal cluster of neurons, where it aids locomotion, whereas FMRF-like neuropeptides, e.g., SDPFLRFamide and GDPFLRFamide, are important cardioactive species that act as neurotransmitters (Linacre et al., 1990). Increasing the spatial resolution in these MSI experiments from molecular mapping of neuronal clusters to the length scale of single neurons opens the possibility of additional insight into neuronal circuits.

## Single Neurons

As neuropeptide profiling by MS made headway from whole CNS to the individual ganglia, single neuron analysis became the new frontier for understanding the functioning of the *L. stagnalis* nervous system. In the early 1990s, microsampling by a focused laser beam became an efficient tool to target the relatively large cells in this organism (Vanveelen et al., 1993; Jimenez et al., 1994). The ability to explore the differences in composition, abundances, and localization of neuropeptides with single cell resolution by MS brought the field to a new level (Li et al., 2000; Rubakhin et al., 2011; Comi et al., 2017; Zhang et al., 2018). In combination with results accumulating from patch clamp studies, a more detailed picture of the biological function of individual neurons has started to emerge.

Prior to MS analysis, neuropeptide expression and localization were tentatively identified throughout neuron clusters and individual neurons in the CNS using in situ hybridization and fluorescent tags. These studies revealed the presence of tetrapeptides (FMRF/FLRF) in multiple behaviorally important networks, such as moto-neurons, whole body withdrawal response moto-neurons, and penis moto-neurons, as well as giant identified neurons (left parietal 1 [LPa1] and right parietal dorsal 1 [RPaD1]) (Bright et al., 1993). Additionally, heptapeptides (GDPFLRF/SDPFLRF) were identified in B group (Bgp) and F group (Fgp) neuron clusters in visceral ganglion (VG). Analysis of the FMRFamide-like neuropeptides using DNA hybridization elucidated a 22 amino acid sequence neuropeptide, SEEPLY. Localization of SEEPLY in the BG of CNS exhibited similar neuronal distributions to the FMRFamide neuropeptide class. The co-expression of the FMRF neuropeptide class and SEEPLY gave rise to interest in more sensitive analytical techniques for neuropeptide mapping (Santama et al., 1993).

One of the first MALDI-MS single neuron studies focused on two neuronal groups, yellow cells and light-yellow cells, and two identified giant neurons, the visceral dorsal 1 (VD1) and right parietal dorsal 2 (RPaD2) (Vanveelen et al., 1993). The yellow cells are located in the right parietal ganglion (RPaG), as well as in the VG. They are known to be involved in the regulation of ion and water metabolism. MADLI-MS analysis of a single yellow cell detected a peptide containing 76 amino acid residues, closely related to the 77-residue sodium influx stimulating peptide (SIS), although this assertion was only supported by the nominal *m/z* 8,891 of the corresponding [M+H]^+^ ion. The light-yellow cells, with a known role in heart modulation, are found in the RPaG and VG. Multiple light-yellow cell peptides (LYCP) were identified based on the nominal *m/z* values of the corresponding ions (Vanveelen et al., 1993; Li et al., 1994b). The VD1 and RPaD2 neurons are located within the VG and RPaG. MALDI-MS analysis showed the presence of α1, α2, and β peptides, where the α peptides were known to modulate heartbeat.

Peptide identification in these early single cell studies could not be purely attributed to the MS results. Nominal mass in itself is insufficient to uniquely identify these large molecules. Invariably, identification relied on known cDNA sequences of the corresponding prohormones and Edman degradation-based sequencing. For example, translating the genetic code to the prepro-SIS protein precursor showed the presence of two components, a signaling peptide and an active 77-residue SIS neuropeptide (see the corresponding entry in Supplementary Table 1). This peptide contained an extra lysine at the carboxyl terminus compared to the 76-residue peptide found by Edman degradation (Vanveelen et al., 1991; de With et al., 1993). Interestingly, the *m/z* 8,891 value detected by MALDI-MS was close to the calculated average *m/z* 8,887 for the protonated 76-residue peptide (Vanveelen et al., 1993). The mass difference of 4 Da between calculated and detected *m/z* in the mass spectrum reflected the limited mass accuracy of the MS instruments at the time. The most important contribution of the MS results in these studies was the information gleaned about the processing of the protein precursors to produce the active peptides.

**FIGURE 4:**
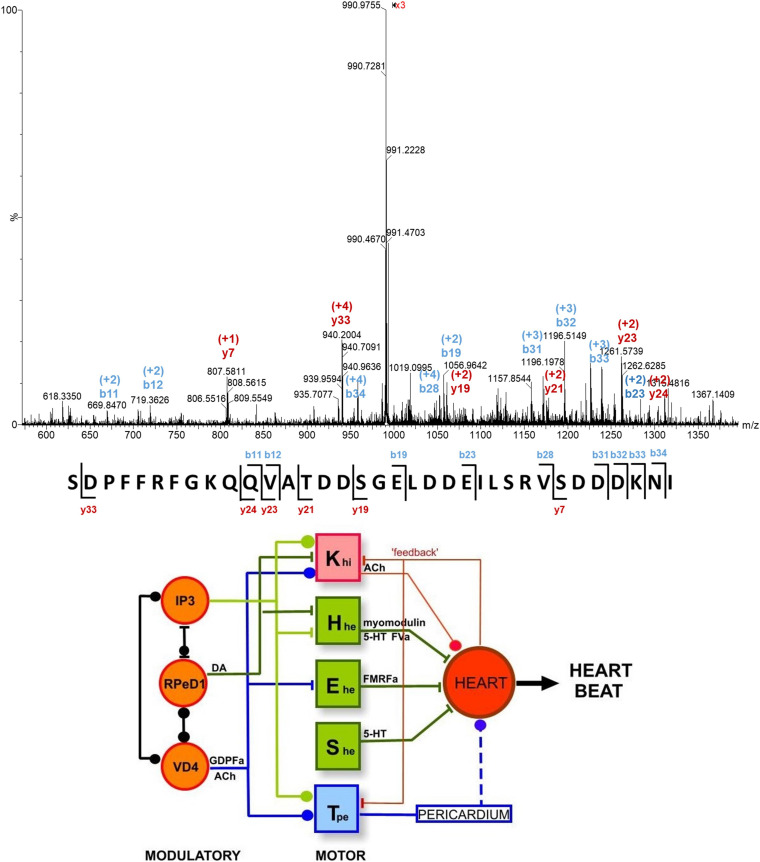
**(Top)** Single cell tandem mass spectrum for a peptide ion with *m/z* 990.468 and a charge state of +4, assigned as a 35-residue acidic peptide, based on its fragmentation pattern and cDNA predicted peptide sequence (Zhang et al., 2018). **(Bottom)** The heartbeat network of *Lymnaea* consists of modulatory and motoneurons connected through excitatory and inhibitory regulation through the release of neuropeptides (Benjamin, 2008). See text for abbreviations.

Significant progress was made when tandem MS became available for single neuron studies. For example, the α1, α2, and β, peptide species that had been detected in VD1 and RPaD2 neurons were verified, and additional peptides were detected in these cells (Jimenez et al., 1998). Some of these peptides were sequenced and assigned as members of the SCP family. More recently, capillary microsampling electrospray ionization (ESI) tandem MS was used to identify the complete primary structure of an unknown 28-residue peptide, and verify the presence of the acidic peptide in the Fgp neuron cluster (see top panel of Figure 4) (Zhang et al., 2018).

Single neuron MS analyses of different neuropeptide classes in combination with patch clamp data enhanced our understanding of the role specific neurons and their neuropeptides play in relation to biological functions. The FMRFamide neuropeptide family was associated with heart modulation, thus their presence in cardio-regulatory neurons was explored. MALDI-MS analysis of an excitatory moto-neuron, E_*he*_, and an E group (Egp) neuron found that these cells expressed tetrapeptides (FMRF/FLRF) from this family, and the peptide pQFLRLI, whereas the visceral white interneuron (VWI) and Bgp single neurons were shown to express the heptapeptides GDPFLRF and SDPFLRF. The dissimilarities found between the cardio-regulatory neurons suggest differences in peptide functions, revealing the necessity of single neuron analysis within the cardio-regulatory system (Vanveelen et al., 1993; Jimenez et al., 1994; Worster et al., 1998; Santama and Benjamin, 2000).

The accumulating compositional data on single neurons taken together with electrophysiological information enabled the identification of neuronal networks responsible for physiological functions, including the networks for heartbeat, whole-body withdrawal, feeding, and respiration (Benjamin, 2008). The heartbeat network of *L. stagnalis* consists of three interconnected modulatory neurons (IP3, RPeD1, and VD4), four excitatory (H_*he*_, E_*he*_, S_*he*_, and T_*pe*_) and one inhibitory (K_*hi*_) moto-neurons (see bottom panel of Figure 4). Excitatory and inhibitory regulation of these neurons and the heart occurs through the release of specific neuropeptides and transmitters. For example, FMRFamide released by the E_*he*_ moto-neuron increases the rate of heartbeat. Conversely, acetylcholine released by the K_*hi*_ moto-neuron inhibits heartbeat.

Another biological function of interest in *L. stagnalis* is gut modulation. Analysis of single giant moto-neuron (B2) in the BG revealed the presence of both SCP A and SCP B peptides. With the use of in situ hybridization followed by MS analysis, it was determined that the SCP neuropeptides have a role in the co-modulation of gut motility (Perry et al., 1999). Analysis of single B2 cells also revealed the presence of four of the five myomodulin neuropeptides: SLSMLRLamide, SMSMLRLamide, PMSMLRLamide, and pQIPMLRLamide. Correlating their presence to their biological role, it was determined that the application of these four neuropeptides modulated contraction frequency and relaxation (Perry et al., 1998).

A uniquely useful tool to explore neuronal circuits and select unidentified neurons for analysis is retrograde tracing. It uses a dye, e.g., nickel-lysine, which is introduced into a neuron in the circuit of interest. The dye is then taken up by the connected neurons, thus labeling them for visual identification and sampling (El Filai et al., 2003). By combining retrograde filling and MALDI-MS analysis, multiple connected neurons from the RPaG were analyzed for their neuropeptide components. It was determined that neurons connected to the male copulation circuit in the RPaG can be divided into two groups: neurons that express FMRF heptapeptides and neurons that do not (El Filali et al., 2015). The combination of retrograde filling with MS technologies is becoming a powerful tool in exploring chemical communication within neuronal circuits.

## Subcellular Localization

Biomolecules can play distinct roles and participate in different sometimes conflicting processes in cells based on their subcellular localization and compartmentalization (Villalobos et al., 2009; Martin, 2010; Diekmann and Pereira-Leal, 2013). With improved sampling techniques and more sensitive mass spectrometers, subcellular MS analysis of organelles, e.g., secretory granules, has become possible (Rubakhin et al., 2000, 2003; Miao et al., 2005). Profiling metabolites and neuropeptides on a subcellular level can face multiple challenges, such as fast molecular turnover rates, low sample volumes, limited amounts of analytes as well as the difficulties of isolating individual compartments with high purity. A novel family of approaches is based on subcellular sampling via laser ablation, microdissection, and extraction through microcapillaries. The combination of microdissection and selective laser ablation revealed large metabolite gradients between the nucleus and the cytoplasm (Stolee et al., 2012). Other studies showed that subcellular compartments can be captured by a nanoelectrospray tip inserted into the cell and the extracted material can be directly electrosprayed (Mizuno et al., 2008), e.g., to study the subcellular metabolism of a drug (Date et al., 2012).

**FIGURE 5:**
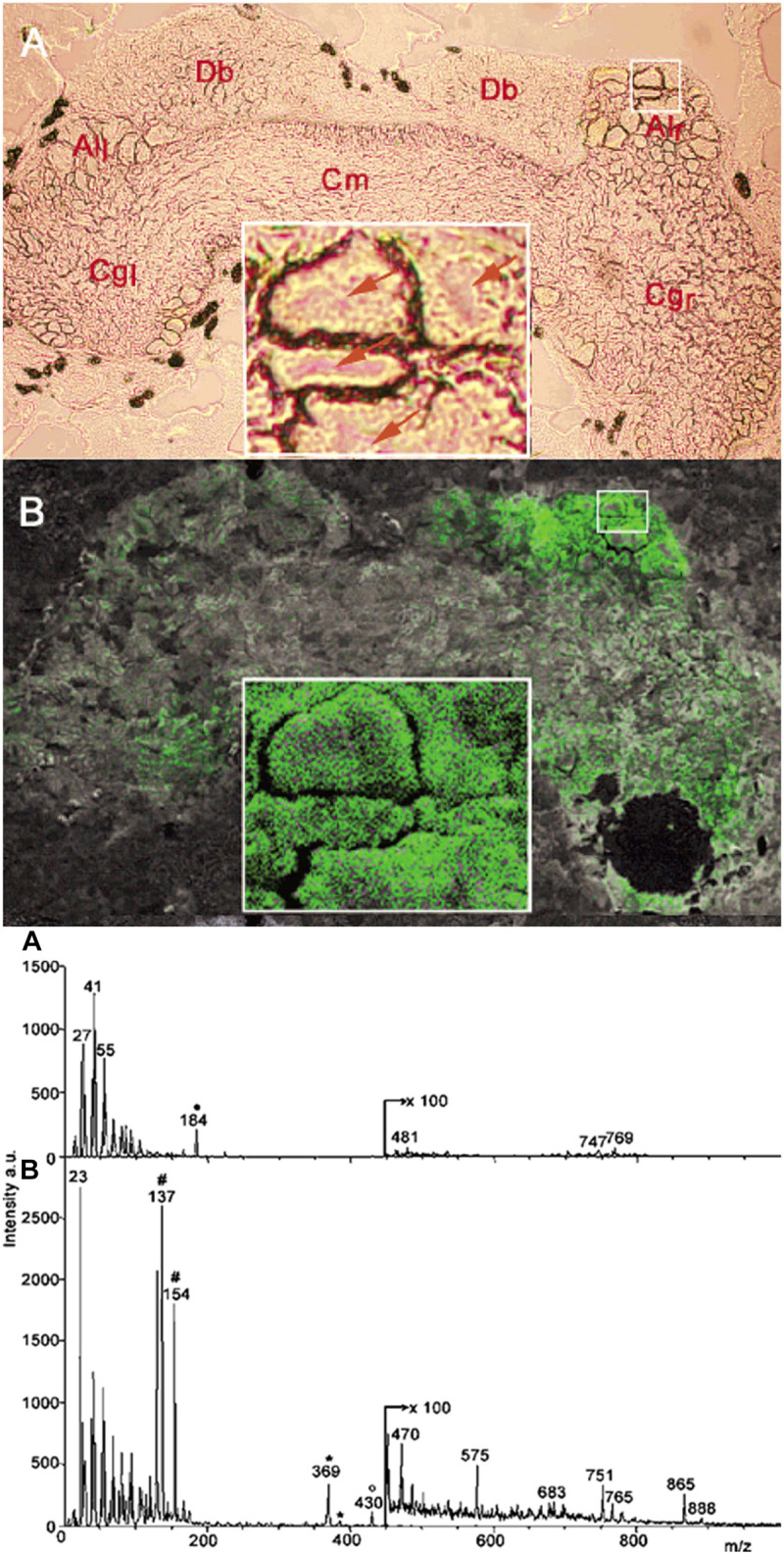
Optical micrograph and ME-SIMS based MSI of cerebral ganglia, and mass spectra of CNS from *L. stagnalis*. Top panel A and B show the morphology and distribution of APGWamide in the left (Cgl) and right (Cgr) ganglia, respectively, with individual neurons in the right anterior lobe (Alr) shown in the insets (Altelaar et al., 2005). Bottom panel A and B compares the SIMS and ME-SIMS mass spectra, respectively. Only the latter detects the protonated APGWamide at *m/z* 430.

The submicrometer spatial resolution of secondary ion MS (SIMS) made this technique uniquely positioned to study subcellular chemical composition. A major obstacle for neuropeptide analysis by SIMS was the significant fragmentation of the produced ions. This problem was alleviated by the introduction of matrix-enhanced SIMS (ME-SIMS) that significantly reduced fragmentation (Altelaar et al., 2005; McDonnell et al., 2005). The method was based on combining the high spatial resolution of SIMS with the organic matrix-based sample preparation techniques in MALDI. Distributions of cholesterol, lipids, and the APGWamide peptide (see Figure 5) within neurons in the cerebral ganglia were captured using ME-SIMS and the mass spectra were compared to MALDI-MS. Results from ME-SIMS exhibited subcellular spatial resolution and the spectra up to m/z 2,500 were consistent with MALDI-MS data.

**FIGURE 6:**
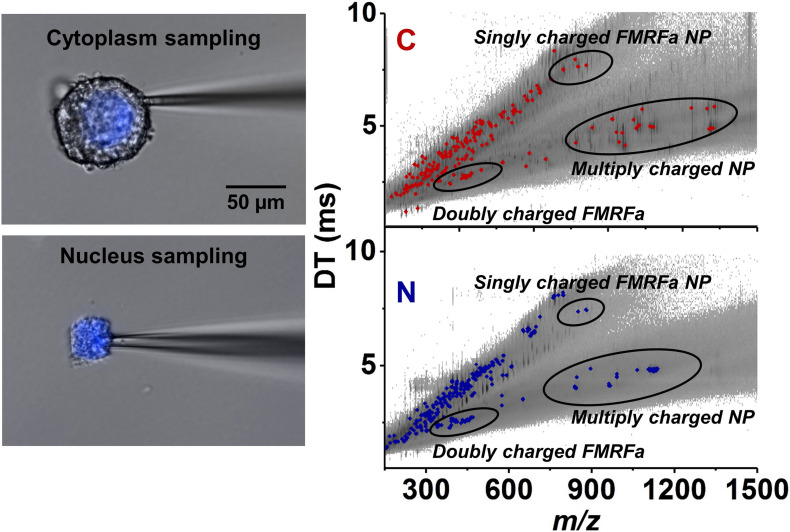
Selective capillary microsampling of cytoplasm (top left panel) and nucleus (bottom left panel) of an Fgp neuron followed by IMS and MS (comparative right panel) (Zhang et al., 2018). The latter reveals the differences in neuropeptide compositions and abundances between the two subcellular compartments.

Capillary microsampling followed by ESI-IMS-MS showed an improved ability to carry out the identification and quantitation of neuropeptides from subcellular samples of *L. stagnalis* neurons (Zhang et al., 2018). Subcellular analysis conducted on Fgp moto-neurons in the visceral ganglion found differences in neuropeptide composition and abundances between the nucleus and the cytoplasm. The nucleus contained six neuropeptides at different abundances within the FMRFamide-like class, whereas the cytoplasm contained nine (see Figure 6). The differences in neuropeptide composition between the nucleus and cytoplasm indicated the presence of active transport, and/or differences in neuropeptide production and degradation rates in each compartment (Zhang et al., 2018).

Comparison of the different techniques and recent improvements in the methods suggest that there is a significant capacity to advance neuropeptide analysis and imaging enabling molecular profiling at the subcellular level.

## Conclusion

The neurons, neuronal circuits, ganglia, and the entire CNS of *L. stagnalis* present an opportunity for MS based exploration of neuropeptide expression and localization. Discovery and profiling of neuropeptides by MS at the single neuron level is facilitated by the relatively large 60 pL to 2 nL soma volumes of their neurons. The new insights on peptide expression are synergistic with the emerging genomic and transcriptomic information, and a wealth of electrophysiological data. Therefore, *L. stagnalis*, next to *A. californica*, remains an important model organism for neuroscience.

Moving forward, a closer integration of genomic information and MS-based peptide discovery is envisioned along the lines of peptidogenomics (Kersten et al., 2011). With the advent of spatially resolved single cell transcriptomics based on multiplexed error-robust fluorescence in-situ hybridization (MERFISH) cell types are identified with unprecedented specificity (Close et al., 2021). Correlations between spatial distributions of transcript-defined cell types and neuropeptides can shed new light complex CNS functions.

Mass spectrometry offers the advantage of untargeted exploration of peptides, ultimately covering the entire peptidome of the CNS (Jimenez et al., 2006; Li and Smit, 2010). A full complement of the expressed peptides and their posttranslational modifications will help to unravel the chemical determinants of the neuronal state. It is expected that sampling protocols and techniques will continue to progress such that single cell and subcellular analysis, and molecular imaging of neuropeptides are refined, and become available to the study of species with more complex CNS.

## Author Contributions

AV and ZP conceived the study, designed the experiments, and conducted the data analysis. EW and LZ raised the snails and performed the dissection of the animals. EW, GM, SS, and LZ conducted the mass spectrometry experiments and evaluated the data. EW, ZP, and AV wrote the manuscript with input from all authors. All authors contributed to the article and approved the submitted version.

## Conflict of Interest

The authors declare that the research was conducted in the absence of any commercial or financial relationships that could be construed as a potential conflict of interest.

## References

[B1] Ahn S. J. Martin R. Rao S. Choi M. Y. (2017). Neuropeptides predicted from the transcriptome analysis of the gray garden slug Deroceras reticulatum. *Peptides* 93 51–65. 10.1016/j.peptides.2017.05.005 28502716

[B2] Altelaar A. F. M. van Minnen J. Jimenez C. R. Heeren R. M. A. Piersma S. R. (2005). Direct molecular Imaging of Lymnaea stagnalis nervous tissue at subcellular spatial resolution by mass spectrometry. *Anal. Chem.* 77 735–741. 10.1021/ac048329g 15679338

[B3] Azevedo F. A. C. Carvalho L. R. B. Grinberg L. T. Farfel J. M. Ferretti R. E. L. Leite R. E. P. (2009). Equal numbers of neuronal and nonneuronal cells make the human brain an isometrically scaled-up primate brain. *J. Comp. Neurol.* 513 532–541. 10.1002/cne.21974 19226510

[B4] Benjamin P. R. (2008). *Lymnaea. Scholarpedia [Online], 3(1).* Available online at: http://www.scholarpedia.org/article/Lymnaea (accessed 24, January, 2021)

[B5] Benjamin P. R. Kemenes I. (2013). *Lymnaea Neuropeptide Genes. Scholarpedia [Online], 8(7).* Available online at: http://www.scholarpedia.org/article/Lymnaea_neuropeptide_genes (accessed 24, January, 2021)

[B6] Benjamin P. R. Kemenes I. (2020). “Peptidergic systems in the pond snail lymnaea: from genes to hormones and behavior,” in *Advances in Invertebrate (Neuro) Endocrinology*, eds Lange A. B. Saleuddin S. Orchard I. (Palm Bay, FL: Apple Academic Press).

[B7] Bogerd J. Li K. W. Jimenez C. R. Vanderschors R. C. Ebberink R. H. M. Geraerts W. P. M. (1994). Processing, axonal-transport and cardioregulatory functions of peptides derived from 2 related prohormones generated by alternative splicing of a single-gene in identified neurons VD1 and RPD2 of Lymnaea. *Mol. Brain Res.* 23, 66–72. 10.1016/0169-328x(94)90212-7 7518031

[B8] Bouetard A. Noirot C. Besnard A. L. Bouchez O. Choisne D. Robe E. (2012). Pyrosequencing-based transcriptomic resources in the pond snail Lymnaea stagnalis, with a focus on genes involved in molecular response to diquat-induced stress. *Ecotoxicology* 21 2222–2234. 10.1007/s10646-012-0977-1 22814884

[B9] Brierley M. J. Yeoman M. S. Benjamin P. R. (1997). Glutamatergic N2v cells are central pattern generator interneurons of the Lymnaea feeding system: new model for rhythm generation. *J. Neurophysiol.* 78 3396–3407. 9405553 10.1152/jn.1997.78.6.3396

[B10] Bright K. Kellett E. Saunders S. E. Brierley M. Burke J. F. Benjamin P. R. (1993). Mutually exclusive expression of alternatively spliced FMRFamide transcripts in identified neuronal systems of the snail Lymnaea. *J. Neurosci.* 13 2719–2729. 8501534 10.1523/JNEUROSCI.13-06-02719.1993 PMC6576478

[B11] Buckett K. J. Peters M. Benjamin P. R. (1990a). Excitation and inhibition of the heart of the snail, Lymnaea, by non-FMRFamidergic motoneurons. *Journal of Neurophysiology* 63 1436–1447. 2358884 10.1152/jn.1990.63.6.1436

[B12] Buckett K. J. Peters M. Dockray G. J. Vanminnen J. Benjamin P. R. (1990b). Regulation of heartbeat in Lymnaea by motoneurons containing FMRFamide-like peptides. *J. Neurophysiol.* 63 1426–1435. 1972741 10.1152/jn.1990.63.6.1426

[B13] Caprioli R. M. Farmer T. B. Gile J. (1997). Molecular imaging of biological samples: localization of peptides and proteins using MALDI-TOF MS. *Anal. Chem.* 69 4751–4760. 10.1021/ac970888i 9406525

[B14] Chaurand P. Stoeckli M. Caprioli R. M. (1999). Direct profiling of proteins in biological tissue sections by MALDI mass spectrometry. *Anal. Chem.* 71 5263–5270. 10.1021/ac990781q 10596208

[B15] Chen R. B. Li L. J. (2010). Mass spectral imaging and profiling of neuropeptides at the organ and cellular domains. *Anal. Bioanal. Chem.* 397 3185–3193. 10.1007/s00216-010-3723-7 20419488 PMC6314848

[B16] Close J. L. Long B. R. Zeng H. (2021). Spatially resolved transcriptomics in neuroscience. *Nat. Methods* 18 23–25. 10.1038/s41592-020-01040-z 33408398

[B17] Comi T. J. Do T. D. Rubakhin S. S. Sweedler J. V. (2017). Categorizing cells on the basis of their chemical profiles: progress in single-cell mass spectrometry. *J. Am. Chem. Soc.* 139 3920–3929. 10.1021/jacs.6b12822 28135079 PMC5364434

[B18] Date S. Mizuno H. Tsuyama N. Harada T. Masujima T. (2012). Direct drug metabolism monitoring in a live single hepatic cell by video mass spectrometry. *Anal. Sci.* 28 201–203. 10.2116/analsci.28.201 22451357

[B19] Davison A. Blaxter M. L. (2005). An expressed sequence tag survey of gene expression in the pond snail Lymnaea stagnalis, an intermediate vector of trematodes [corrected]. *Parasitology* 130(Pt 5) 539–552. 10.1017/s0031182004006791 15991497

[B20] De Boer P. Ter Maat A. Pieneman A. W. Croll R. P. Kurokawa M. Jansen R. F. (1997). Functional role of peptidergic anterior lobe neurons in male sexual behavior of the snail Lymnaea stagnalis. *J. Neurophysiol.* 78 2823–2833. 9405503 10.1152/jn.1997.78.6.2823

[B22] de With N. D. van der Schors R. C. Boer H. H. Ebberink R. H. M. (1993). The sodium influx stimulating peptide of the pulmonate freshwater snail Lymnaea stagnalis. *Peptides* 14 783–789. 10.1016/0196-9781(93)90114-V 8234026

[B23] Dewith N. D. Vanderschors R. C. (1992). SKPYMRFamide, a novel FMRFamide-related peptide in the snail Lymnaea-stagnalis. *Neuroreport* 3 612–614. 10.1097/00001756-199207000-00017 1421117

[B24] Diekmann Y. Pereira-Leal J. B. (2013). Evolution of intracellular compartmentalization. *Biochem. J.* 449 319–331. 10.1042/bj20120957 23240612

[B25] Dreisewerd K. Kingston R. Geraerts W. P. M. Li K. W. (1997). Direct mass spectrometric peptide profiling and sequencing of nervous tissues to identify peptides involved in male copulatory behavior in Lymnaea stagnalis. *Int. J. Mass Spectr.* 169 291–299. 10.1016/s0168-1176(97)00220-6

[B26] Ebberink R. H. M. Price D. A. Vanloenhout H. Doble K. E. Riehm J. P. Geraerts W. P. M. (1987). The brain of Lymnaea contains a family of FMRFamide-like peptides. *Peptides* 8 515–522. 10.1016/0196-9781(87)90018-0 3658814

[B27] El Filai Z. Hornshaw M. Smit A. B. Li K. W. (2003). Retrograde labeling of single neurons in conjunction with MALDI high-energy collision-induced dissociation MS/MS analysis for peptide profiling and structural characterization. *Anal. Chem.* 75 2996–3000. 10.1021/ac034057q 12964743

[B28] El Filali Z. de Boer P. Pieneman A. W. de Lange R. P. J. Jansen R. F. Ter Maat A. (2015). Single-cell analysis of peptide expression and electrophysiology of right parietal neurons involved in male copulation behavior of a simultaneous hermaphrodite. *Invertebrate Neurosci.* 15:7. 10.1007/s10158-015-0184-x 26639152 PMC4670828

[B29] El Filali Z. Van Minnen J. Liu W. K. Smit A. B. Li K. W. (2006). Peptidomics analysis of neuropeptides involved in copulatory behavior of the mollusk Lymnaea stagnalis. *J. Proteome Res.* 5 1611–1617. 10.1021/pr060014p 16823968

[B30] Feng Z. P. Zhang Z. van Kesteren R. E. Straub V. A. van Nierop P. Jin K. (2009). Transcriptome analysis of the central nervous system of the mollusc Lymnaea stagnalis. *BMC Genomics* 10:451. 10.1186/1471-2164-10-451 19775440 PMC2760584

[B31] Fodor I. Hussein A. A. A. Benjamin P. R. Koene J. M. Pirger Z. (2020a). The unlimited potential of the great pond snail. *Lymnaea stagnalis*. *Elife* 9:e56962. 10.7554/eLife.56962 32539932 PMC7297532

[B32] Fodor I. Svigruha R. Kemenes G. Kemenes I. Pirger Z. (2021). The great pond snail (Lymnaea stagnalis) as a model of ageing and age-related memory impairment: an overview. *J. Gerontol A Biol. Sci. Med. Sci.* 10.1093/gerona/glab014 [Epup ahead of print], 33453110

[B33] Fodor I. Urban P. Scott A. P. Pirger Z. (2020b). A critical evaluation of some of the recent so-called ‘evidence’ for the involvement of vertebrate-type sex steroids in the reproduction of mollusks. *Mol. Cell. Endocrinol.* 516:110949. 10.1016/j.mce.2020.110949 32687858

[B34] Fulton D. Kemenes I. Andrew R. J. Benjamin P. R. (2005). A single time-window for protein synthesis-dependent long-term memory formation after one-trial appetitive conditioning. *Eur. J. Neurosci.* 21 1347–1358. 10.1111/j.1460-9568.2005.03970.x 15813944

[B35] Hoek R. M. Li K. W. van Minnen J. Lodder J. C. de Jong-Brink M. Smit A. B. (2005). LFRFamides: a novel family of parasitation-induced -RFamide neuropeptides that inhibit the activity of neuroendocrine cells in Lymnaea stagnalis. *J. Neurochem.* 92 1073–1080. 10.1111/j.1471-4159.2004.02927.x 15715658

[B36] Hummon A. B. Amare A. Sweedler J. V. (2006). Discovering new invertebrate neuropeptides using mass spectrometry. *Mass Spectr. Rev.* 25 77–98. 10.1002/mas.20055 15937922

[B37] Hummon A. B. Sweedler J. V. Corbin R. W. (2003). Discovering new neuropeptides using single-cell mass spectrometry. *Trac-Trends Anal. Chem.* 22 515–521. 10.1016/s0165-9936(03)00901-4

[B38] Jimenez C. R. Li K. W. Dreisewerd K. Spijker S. Kingston R. Bateman R. H. (1998). Direct mass spectrometric peptide profiling and sequencing of single neurons reveals differential peptide patterns in a small neuronal network. *Biochemistry* 37 2070–2076. 10.1021/bi971848b 9485334

[B39] Jimenez C. R. Spijker S. de Schipper S. Lodder J. C. Janse C. K. Geraerts W. P. M. (2006). Peptidomics of a single identified neuron reveals diversity of multiple neuropeptides with convergent actions on cellular excitability. *J. Neurosci.* 26 518–529. 10.1523/jneurosci.2566-05.2006 16407549 PMC6674408

[B40] Jimenez C. R. ter Maat A. Pieneman A. Burlingame A. L. Smit A. B. Li K. W. (2004). Spatio-temporal dynamics of the egg-laying-inducing peptides during an egg-laying cycle: a semiquantitative matrix-assisted laser desorption/ionization mass spectrometry approach. *J. Neurochem.* 89 865–875. 10.1111/j.1471-4159.2004.02353.x 15140186

[B41] Jimenez C. R. Vanveelen P. A. Li K. W. Wildering W. C. Geraerts W. P. M. Tjaden U. R. (1994). Neuropeptide expression and processing as revealed by direct matrix-assisted laser-desorption ionization mass-spectrometry of single neurons. *J. Neurochem.* 62 404–407. 8263544 10.1046/j.1471-4159.1994.62010404.x

[B42] Kellett E. Perry S. J. Santama N. Worster B. M. Benjamin P. R. Burke J. F. (1996). Myomodulin gene of Lymnaea: structure, expression, and analysis of neuropeptides. *J. Neurosci.* 16 4949–4957. 8756426 10.1523/JNEUROSCI.16-16-04949.1996 PMC6579316

[B43] Kellett E. Saunders S. E. Li K. W. Staddon J. W. Benjamin P. R. Burke J. F. (1994). Genomic organization of the FMRFamide gene in Lymnaea - multiple exons encoding novel neuropeptides. *J. Neurosci.* 14 6564–6570. 7965060 10.1523/JNEUROSCI.14-11-06564.1994 PMC6577258

[B44] Kemenes G. Staras K. Benjamin P. R. (2001). Multiple types of control by identified interneurons in a sensory-activated rhythmic motor pattern. *J. Neurosci.* 21 2903–2911. 10.1523/jneurosci.21-08-02903.2001 11306642 PMC6762524

[B45] Kersten R. D. Yang Y. L. Xu Y. Q. Cimermancic P. Nam S. J. Fenical W. (2011). A mass spectrometry-guided genome mining approach for natural product peptidogenomics. *Nat. Chem. Biol.* 7 794–802. 10.1038/nchembio.684 21983601 PMC3258187

[B46] Kiss T. Pirger Z. (2006). *Neuropeptides as Modulators and Hormones in Terrestrial Snails: Their Occurence, Distribution and Physiological Significance.* Kerala: Transworld Research Network.

[B47] Koene J. M. (2010). Neuro-endocrine control of reproduction in hermaphroditic freshwater snails: mechanisms and evolution. *Front. Behav. Neurosci.* 4:167. 10.3389/fnbeh.2010.00167 21088700 PMC2981420

[B48] Kojima S. Sunada H. Mita K. Sakakibara M. Lukowiak K. Ito E. (2015). Function of insulin in snail brain in associative learning. *J. Comp. Physiol. Neuroethol. Sensory Neural Behav. Physiol.* 201 969–981. 10.1007/s00359-015-1032-5 26233474

[B50] Li K. W. Van Minnen J. Van Veelen P. A. Van der Greef J. Geraerts W. P. M. (1996). Structure, localization and action of a novel inhibitory neuropeptide involved in the feeding of Lymnaea. *Mol. Brain Res.* 37, 267–272. 10.1016/0169-328x(95)00333-n 8738160

[B51] Li K. W. Jimenez C. R. Vanveelen P. A. Geraerts W. P. M. (1994a). Processing and targeting of a molluscan egg-laying peptide prohormone as revealed by mass-spectrometric peptide fingerprinting and peptide sequencing. *Endocrinology* 134 1812–1819. 10.1210/en.134.4.1812 8137747

[B52] Li K. W. Kingston R. Dreisewerd K. Jimenez C. R. vanderSchors R. C. Bateman R. H. (1997). Structural elucidation of a peptide from a single neuron by matrix-assisted laser desorption/ionization employing a tandem double focusing magnetic-orthogonal acceleration time-of-flight mass spectrometer. *Anal. Chem.* 69 563–565. 10.1021/ac9609440

[B53] Li K. W. Smit A. B. (2010). “Mass spectrometric analysis of molluscan neuropeptides,” in *Peptidomics: Methods and Protocols*, ed. Soloviev M. (Totowa, NJ: Humana Press), 49–56. 10.1007/978-1-60761-535-4_4 20013199

[B54] Li K. W. Vangolen F. A. Vanminnen J. Vanveelen P. A. Vandergreef J. Geraerts W. P. M. (1994b). Structural identification, neuronal synthesis, and role in male copulation of myomodulin-a of Lymnaea - a study involving direct peptide profiling of nervous-tissue by mass-spectrometry. *Mol. Brain Res.* 25 355–358. 10.1016/0169-328x(94)90172-4 7808235

[B55] Li L. J. Garden R. W. Sweedler J. V. (2000). Single-cell MALDI: a new tool for direct peptide profiling. *Trends Biotechnol.* 18 151–160. 10.1016/s0167-7799(00)01427-x 10740261

[B56] Linacre A. Kellett E. Saunders S. Bright K. Benjamin P. R. Burke J. F. (1990). Cardioactive neuropeptide PHE-MET-ARG-PHE-NH2 (FMRFamide) and novel related peptides are encoded in multiple copies by a single gene in the snail Lymnaea-stagnalis. *J. Neurosci.* 10 412–419. 1968092 10.1523/JNEUROSCI.10-02-00412.1990 PMC6570152

[B57] Martin W. (2010). Evolutionary origins of metabolic compartmentalization in eukaryotes. *Philos. Trans. R. Soc. B Biol. Sci.* 365 847–855. 10.1098/rstb.2009.0252 20124349 PMC2817231

[B58] McDonnell L. A. Piersma S. R. Altelaar A. F. M. Mize T. H. Luxembourg S. L. Verhaert P. (2005). Subcellular imaging mass spectrometry of brain tissue. *J. Mass Spectr.* 40 160–168. 10.1002/jms.735 15706616

[B59] Miao H. Rubakhin S. S. Sweedler J. V. (2005). Subcellular analysis of D-Aspartate. *Anal. Chem.* 77 7190–7194. 10.1021/ac0511694 16285665

[B60] Mizuno H. Tsuyama N. Harada T. Masujima T. (2008). Live single-cell video-mass spectrometry for cellular and subcellular molecular detection and cell classification. *J. Mass Spectr.* 43 1692–1700. 10.1002/jms.1460 18615771

[B61] Nagle G. T. de Jong-Brink M. Painter S. D. Li K. W. (2001). Structure, localization and potential role of a novel molluscan trypsin inhibitor in Lymnaea. *Eur. J. Biochem.* 268, 1213–1221. 10.1046/j.1432-1327.2001.01972.x 11231272

[B62] Patel B. A. Arundell M. Parker K. H. Yeoman M. S. O’Hare D. (2005). Simple and rapid determination of serotonin and catecholamines in biological tissue using high-performance liquid chromatography with electrochemical detection. *J. Chromatogr. B Anal. Technol. Biomed. Life Sci.* 818 269–276. 10.1016/j.jchromb.2005.01.008 15734169

[B63] Perry S. J. Dobbins A. C. Schofield M. G. Piper M. R. Benjamin P. R. (1999). Small cardioactive peptide gene: structure, expression and mass spectrometric analysis reveals a complex pattern of co-transmitters in a snail feeding neuron. *Eur. J. Neurosci.* 11 655–662. 10051766 10.1046/j.1460-9568.1999.00472.x

[B64] Perry S. J. Straub V. A. Kemenes G. Santama N. Worster B. M. Burke J. F. (1998). Neural modulation of gut motility by myomodulin peptides and acetylcholine in the snail Lymnaea. *J. Neurophysiol.* 79 2460–2474. 9582220 10.1152/jn.1998.79.5.2460

[B65] Pirger Z. Naskar S. Laszlo Z. Kemenes G. Reglodi D. Kemenes I. (2014). Reversal of age-related learning deficiency by the vertebrate PACAP and IGF-1 in a novel invertebrate model of aging: the pond snail (Lymnaea stagnalis). *J. Gerontol. A Biol. Sci. Med. Sci.* 69 1331–1338. 10.1093/gerona/glu068 24846768 PMC4197904

[B66] Rivi V. Benatti C. Colliva C. Radighieri G. Brunello N. Tascedda F. (2020). Lymnaea stagnalis as model for translational neuroscience research: From pond to bench. *Neurosci. Biobehav. Rev.* 108 602–616. 10.1016/j.neubiorev.2019.11.020 31786320

[B67] Rubakhin S. S. Garden R. W. Fuller R. R. Sweedler J. V. (2000). Measuring the peptides in individual organelles with mass spectrometry. *Nat. Biotechnol.* 18 172–175. 10.1038/72622 10657123

[B68] Rubakhin S. S. Greenough W. T. Sweedler J. V. (2003). Spatial profiling with MALDI MS: distribution of neuropeptides within single neurons. *Anal. Chem.* 75 5374–5380. 10.1021/ac034498 14710814

[B69] Rubakhin S. S. Romanova E. V. Nemes P. Sweedler J. V. (2011). Profiling metabolites and peptides in single cells. *Nat. Methods* 8 S20–S29. 10.1038/nmeth.1549 21451513 PMC3312877

[B70] Sadamoto H. Takahashi H. Okada T. Kenmoku H. Toyota M. Asakawa Y. (2012). De novo sequencing and transcriptome analysis of the central nervous system of mollusc Lymnaea stagnalis by deep RNA sequencing. *PLoS One* 7:e42546. 10.1371/journal.pone.0042546 22870333 PMC3411651

[B71] Santama N. Benjamin P. R. (2000). Gene expression and function of FMRFamide-related neuropeptides in the snail Lymnaea. *Microscopy Res. Tech.* 49 547–556. 10.1002/1097-0029(20000615)49:6<547::aid-jemt5<3.0.co;2-y 10862111

[B72] Santama N. Brierley M. Burke J. F. Benjamin P. R. (1994a). Neural-network controlling feeding in Lymnaea-stagnalis - immunocytochemical localization of myomodulin, small cardioactive peptide, buccalin, and FMRFamide-related peptides. *J. Comp. Neurol.* 342 352–365. 10.1002/cne.903420304 7912700

[B73] Santama N. Li K. W. Bright K. E. Yeoman M. Geraerts W. P. M. Benjamin P. R. (1993). Processing of the FMRFamide precursor protein in the snail Lymnaea-stagnalis - characterization and neuronal localization of a novel peptide, SEEPLY. *Eur. J. Neurosci.* 5 1003–1016. 10.1111/j.1460-9568.1993.tb00952.x 7904219

[B74] Santama N. Wheeler C. H. Burke J. F. Benjamin P. R. (1994b). Neuropeptides myomodulin, small cardioactive peptide, and buccalin in the central-nervous-system of Lymnaea-stagnalis - purification, immunoreactivity, and artifacts. *J. Comp. Neurol.* 342 335–351. 10.1002/cne.903420303 8021339

[B75] Santama N. Wheeler C. H. Skingsley D. R. Yeoman M. S. Bright K. Kaye I. (1995). Identification, distribution and physiological-activity of 3 novel neuropeptides of Lymnaea - EFLRLamide and PQFYRLamide encoded by the FMRFamide gene, and a related peptide. *Eur. J. Neurosci.* 7 234–246. 10.1111/j.1460-9568.1995.tb01059.x 7757260

[B76] Saunders S. E. Bright K. Kellett E. Benjamin P. R. Burke J. F. (1991). Neuropeptides GLY-ASP-PRO-PHE-LEU-ARG-PHE-amide (GDPFLRFamide) and SERASP-PRO-PHE-LEU-ARG-PHE-amide (SDPFLRFamide) are encoded by an exon 3’ to PHE-MET-ARG-PHE-NH2 (FMRFamide) in the snail Lymnaea-stagnalis. *J. Neurosci.* 11, 740–745. 2002360 10.1523/JNEUROSCI.11-03-00740.1991 PMC6575343

[B77] Schot L. P. C. Boer H. H. Swaab D. F. Vannoorden S. (1981). Immuno-cytochemical demonstration of peptidergic neurons in the central nervous-system of the pond snail lymnaea-stagnalis with antisera raised to biologically-active peptides of vertebrates. *Cell Tissue Res.* 216 273–291. 10.1007/bf00233620 6112067

[B78] Smit A. B. Geraerts W. P. M. Meester I. Vanheerikhuizen H. Joosse J. (1991). Characterization of a cdna clone encoding molluscan insulin-related peptide-ii of lymnaea-stagnalis. *Eur. J. Biochem.* 199 699–703. 10.1111/j.1432-1033.1991.tb16173.x 1868853

[B79] Smit A. B. Jimenez C. R. Dirks R. W. Croll R. P. Geraerts W. P. M. (1992). Characterization of a cDNA clone encoding multiple copies of the neuropeptide APGWamide in the mollusk Lymnaea-stagnalis. *J. Neurosci.* 12 1709–1715. 1578265 10.1523/JNEUROSCI.12-05-01709.1992 PMC6575885

[B80] Smit A. B. Spijker S. Van Minnen J. Burke J. F. De Winter F. Van Elk R. (1996). Expression and characterization of molluscan insulin-related peptide VII from the molluscLymnaea stagnalis. *Neuroscice* 70, 589–596. 10.1016/0306-4522(95)00378-9 8848162

[B81] Smit A. B. Thijsen S. F. T. Geraerts W. P. M. (1993). cDNA cloning of the sodium-influx-stimulating peptide in the mollusc, Lymnaea stagnalis. *Eur. J. Biochem.* 215, 397–400. 10.1111/j.1432-1033.1993.tb18046.x 8344306

[B82] Smit A. B. van Kesteren R. E. Spijker S. Van Minnen J. van Golen F. A. Jimenez C. R. (2003). Peptidergic modulation of male sexual behavior in Lymnaea stagnalis: structural and functional characterization of -FVamide neuropeptides. *J. Neurochem.* 87 1245–1254. 10.1046/j.1471-4159.2003.02086.x 14622104

[B83] Stevens L. Kumar S. Blaxter L. M. (2016). *Lymnaea Stagnalis, Whole Genome Shotgun Sequencing Project.*

[B84] Stolee J. A. Shrestha B. Mengistu G. Vertes A. (2012). Observation of Subcellular Metabolite Gradients in Single Cells by Laser Ablation Electrospray Ionization Mass Spectrometry. *Angewandte Chemie Int. Edn.* 51 10386–10389. 10.1002/anie.201205436 22952015

[B85] Sweedler J. V. Li L. Rubakhin S. S. Alexeeva V. Dembrow N. C. Dowling O. (2002). Identification and characterization of the feeding circuit-activating peptides, a novel neuropeptide family of Aplysia. *J. Neurosci.* 22 7797–7808. 12196603 10.1523/JNEUROSCI.22-17-07797.2002 PMC6757975

[B86] Tensen C. P. Cox K. J. A. Smit A. B. van der Schors R. C. Meyerhof W. Richter D. (1998). The Lymnaea cardioexcitatory peptide (LyCEP) receptor: a G-proteincoupled receptor for a novel member of the RFamide neuropeptide family. *J. Neurosci.* 18, 9812–9821. 9822740 10.1523/JNEUROSCI.18-23-09812.1998 PMC6793288

[B87] Totani Y. Aonuma H. Oike A. Watanabe T. Hatakeyama D. Sakakibara M. (2019). Monoamines, insulin and the roles they play in associative learning in pond snails. *Front. Behav. Neurosci.* 13:65. 10.3389/fnbeh.2019.00065 31001093 PMC6454038

[B88] van Tol-Steye H. Lodder J. C. Mansvelder H. D. Planta R. J. van Heerikhuizen H. Kits K. S. (1999). Roles of G-protein beta gamma, arachidonic acid, and phosphorylation in convergent activation of an S-like potassium conductance by dopamine, Ala-Pro-Gly-Trp-NH2, and Phe-Met-Arg-Phe-NH2. *J. Neurosci.* 19 3739–3751. 10234006 10.1523/JNEUROSCI.19-10-03739.1999 PMC6782690

[B89] Van Kesteren R. Smit A. De Lange R. Kits K. Van Golen F. Van Der Schors R. (1995). Structural and functional evolution of the vasopressin/oxytocin superfamily: vasopressin-related conopressin is the only member present in Lymnaea, and is involved in the control of sexual behavior. *J. Neurosci.* 15, 5989–5998. 10.1523/jneurosci.15-09-05989.1995 7666183 PMC6577683

[B90] Van Kesteren R. E. Carter C. Dissel H. M. G. van Minnen J. Gouwenberg Y. Syed N. I. (2006). Local synthesis of actin-binding protein β-thymosin regulates neurite outgrowth. *J. Neurosci.* 26, 152–157. 10.1523/jneurosci.4164-05.2006 16399682 PMC6674307

[B91] van Kesteren R. E. Smit A. B. Dirks R. W. de With N. D. Geraerts W. P. Joosse J. (1992). Evolution of the vasopressin/oxytocin superfamily: characterization of a cDNA encoding a vasopressin-related precursor, preproconopressin, from the mollusc Lymnaea stagnalis. *Proc. Natl. Acad. Sci.* 89, 4593–4597. 10.1073/pnas.89.10.4593 1584795 PMC49129

[B92] Vanveelen P. A. Jimenez C. R. Li K. W. Wildering W. C. Geraerts W. P. M. Tjaden U. R. (1993). Direct peptide profiling of single neurons by matrix-assisted laser-desorption ionization mass-spectrometry. *Organic Mass Spectr.* 28 1542–1546. 10.1002/oms.1210281229

[B93] Vanveelen P. A. Tjaden U. R. Vandergreef J. Dewith N. D. (1991). Sequence-informative fragmentation in an 8.9 kda oligopeptide using plasma desorption mass-spectrometry. *Organic Mass Spectr.* 26 345–346. 10.1002/oms.1210260429

[B94] Villalobos C. Alonso M. T. Garcia-Sancho J. (2009). “Bioluminescence imaging of calcium oscillations inside intracellular organelles,” in *Bioluminescence: Methods and Protocols*, Second Edn, eds Rich P. B. Douillet C. (Totowa, NJ: Humana Press), 203–214. 10.1007/978-1-60327-321-3_17 19685311

[B95] Wang Y. Wang M. Yin S. Jang R. Wang J. Xue Z. (2015). NeuroPep: a comprehensive resource of neuropeptides. *Database (Oxford)* 2015:bav038. 10.1093/database/bav038 PMC4414954 25931458

[B96] Weiss S. Goldberg J. I. Chohan K. S. Stell W. K. Drummond G. I. Lukowiak K. (1984). Evidence for FMRFamide as a neurotransmitter in the gill of Aplysia-californica. *J. Neurosci.* 4 1994–2000. 6147396 10.1523/JNEUROSCI.04-08-01994.1984 PMC6564954

[B97] Worster B. M. Yeoman M. S. Benjamin P. R. (1998). Matrix-assisted laser desorption/ionization time of flight mass spectrometric analysis of the pattern of peptide expression in single neurons resulting from alternative mRNA splicing of the FMRFamide gene. *Eur. J. Neurosci.* 10 3498–3507. 10.1046/j.1460-9568.1998.00361.x 9824463

[B98] Zhang L. W. Khattar N. Kemenes I. Kemenes G. Zrinyi Z. Pirger Z. (2018). Subcellular peptide localization in single identified neurons by capillary microsampling mass spectrometry. *Sci. Rep.* 8:12227. 10.1038/s41598-018-29704-z 30111831 PMC6093924

